# Causal inference and risk prediction of gestational diabetes mellitus based on case–control study and Mendel randomization

**DOI:** 10.3389/fnut.2025.1665813

**Published:** 2025-11-03

**Authors:** Ruiqi Li, Yan Sun, Qiulian Liang, Xinyu He, Lijie Nie, Jian Huang, Xiangyuan Yu

**Affiliations:** ^1^The School of Public Health, Guilin Medical University, Guilin, China; ^2^Institute of Biomedical Research, School of Intelligent Medicine and Biotechnology, Guilin Medical University, Guilin, China; ^3^Guangxi Key Laboratory of Diabetic Systems Medicine, Guilin Medical University, Guilin, China

**Keywords:** gestational diabetes mellitus, genetic variant, Mendelian randomization, causal factors, nomogram model

## Abstract

**Aims:**

To evaluate the causal determinants and their risk predictive efficacy of gestational diabetes mellitus (GDM) in Chinese population.

**Methods:**

Genotyping data for candidate genetic variants were collected from 554 cases of GDM and 641 pregnant women with normal glucose tolerance. The associations between these variants and GDM risk were evaluated with the odds ratios (ORs) and their corresponding 95% confidence intervals (CIs). Multivariate Mendelian randomization (MVMR) was employed to validate the GDM causal factors. Subsequently, a GDM early risk prediction nomogram model was developed based on the key clinical and genetic factors identified.

**Results:**

After adjusting age and pre-pregnancy BMI (pre-BMI), the rs6127416 variant showed a significant association with susceptibility to GDM. Comparing the AA genotype to the TT genotype, the adjusted odds ratio (OR) was 2.20 (95%CI = 1.53–3.18, *p* < 0.001), and comparing AA to TT/TA genotypes, the adjusted OR was 2.35 (95%CI = 1.68–3.30, *p* < 0.001). MVMR analysis confirmed the positive causal effects of pre-BMI and fasting plasma glucose (FPG) on GDM (pre-BMI-OR_MVMR_ = 1.80, FPG-OR_MVMR_ = 12.37, *p* < 0.001). A nomogram risk predictive model incorporating pre-BMI, FPG, and rs6127416 demonstrated an area under the ROC curve of 0.808.

**Conclusion:**

Pre-BMI and FPG were determined to be causal factors linked to GDM. The prediction model constructed using key clinical and genetic variables (such as rs6127416-preBMI-FPG) holds promising utility for personalized risk assessment of GDM in the initial trimester of pregnancy, with potential to support early identification of high-risk women and facilitate timely lifestyle or clinical interventions during antenatal care.

## Introduction

Gestational diabetes (GDM) is a pregnancy-related metabolic disorder characterized by glucose intolerance leading to varying degrees of hyperglycemia first detected during pregnancy (1). It affects an estimated 2–20% of pregnant women globally, with a prevalence of 14.8% in China (2). GDM is associated with numerous adverse outcomes for both the mother and the fetus, such as polyhydramnios, infection, ketoacidosis, gestational hypertension, spontaneous abortion, pre-eclampsia, preterm birth, small or large for gestational age, fetal macrosomia, shoulder dystocia, and even stillbirth (3, 4). Following delivery, most women with GDM typically revert to normal glucose metabolism, yet they face an increased risk of developing type 2 diabetes (T2DM) and cardiovascular ailments later in life, as do their offspring (5).

Studies have indicated that the primary pathogenic mechanisms observed in GDM may overlap with those present in T2DM, including insulin resistance, impaired insulin secretion, and aberrant glucose utilization (6, 7). Currently, the etiology of GDM is understood to be multifactorial, involving factors such as advanced maternal age, overweight, obesity, ethnicity, hypertension, and a family history of GDM or T2DM (6, 8). Research indicates that a family history of GDM or T2DM is associated with a higher incidence of GDM, with approximately 32.9% of women with a diabetes family history experiencing GDM, which is about three times higher than those without such a history (9, 10). These findings underscore the significant contribution of genetic factors to the pathogenesis of GDM.

Single nucleotide polymorphisms (SNPs) represent the most prevalent form of genetic variations in the human genome (11). These variations are distributed across various genomic regions, including exons, introns, promoters, enhancers, among others. Thus, an SNP in coding region may affect the amino acid sequence of the encoded protein, while an intronic SNP could affect splicing, and an SNP in promoters might alter gene expression (12, 13). Genome-wide association studies (GWAS), which assess allele frequency differences of genetic variants to uncover genotype–phenotype relationships, have yielded valuable genetic insights into the pathogenesis of human disease (14). To date, numerous SNPs with susceptibility to GDM have been identified. Our previous studies have substantiated a set of SNPs, including those in the *ACE2* gene (rs6632677 and rs2074192), *RXR-γ* gene (rs2134095), *XAB2* gene (rs3760675), *ERBB4* gene (rs1595066), which exhibit significant interactions with pre pregnancy body mass index (pre-BMI), fasting blood glucose (FPG), and glycated hemoglobin (HbA1c), and impact gene expression levels. These SNPs have been notably linked to the risk of GDM (15–18). With the ongoing discovery and validation of associations between susceptibility genes and their SNPs in complex diseases, SNP- based genetic susceptibility research holds promise for elucidating the risk and etiological mechanisms of conditions like GDM.

Mendelian randomization (MR) has emerged as a prominent epidemiological approach for investigating potential causal relationships. This method utilizes SNPs as instrumental variables (IVs) to evaluate causal effects between modifiable non-genetic exposure factors and outcomes (19). The key strength of MR lies in its ability to minimize confounding effects and effectively address reverse causality due to the fixed nature of gene sequence (20). Consequently, the application of MR allows for the development of a nomogram for predicting GDM risk prediction based on robust causal inferences. The nomogram calculates a cumulative score based on the quantitative value of each influencing factor, determined by the magnitude of the regression coefficient, to assess the likelihood or risk of the event. By transforming the predicted probability of relevant outcome events into a visual representation, the nomogram facilitates easier interpretation and comprehension of the results (21, 22). This nomogram model has the potential to identify high-risk pregnant women with GDM at an early stage and facilitate the implementation of targeted prevention and management strategies accordingly (23).

In this study, we aim to undertake a comprehensive case–control study on a large scale to validate the relationship between genetic variants identified through GWAS and the occurrence of GDM in the southern Chinese population. We intend to develop an integrated personalized nomogram model by amalgamating significantly associated SNPs with clinically confirmed genetic causal factors through MR. This model will be utilized for early pregnancy risk prediction of GDM in women.

## Materials and methods

### Study population

In the initial discovery phase, a genome-wide association study (GWAS) was conducted to identify GDM-associated SNPs (GDM-SNPs). The study included 96 GDM patients and 96 age- and pre-BMI-matched healthy pregnant women, all recruited during the same period, using the Infinium Asian Screening Array (ASA, Illumina) BeadChip.

For the validation phase, a total of 1,195 singleton pregnant women (554 GDM patients and 641 healthy controls) with similar characteristics were enrolled at the Affiliated Hospital of Guilin Medical University between September 2014 and April 2016 for genotyping of candidate SNPs. GDM was based on the criteria of the 75 g oral glucose tolerance test (OGTT) conducted at 24–28 weeks of gestation, with fasting plasma glucose (FPG) ≥ 5.1 mmol/L or 1-h plasma glucose (1hPG) ≥ 10.0 mmol/L, or 2hPG ≥ 8.5 mmol/L as per the guidelines established by the International Association of Diabetes and Pregnancy Study Group (IADPSG) (24).

Subjects meeting the following inclusion criteria were enrolled: residency in the Guilin area for over 2 years, singleton pregnancy, and absence of close familial ties. Exclusion criteria encompassed pregnancies with endocrine disorders, severe systemic illnesses, a history of pre-existing type 1 or type 2 diabetes mellitus, or prolonged use of medications affecting glucose metabolism prior to pregnancy. A flow diagram detailing the participant inclusion process has been provided as Supplementary Figure 1. Approval of this research protocol was obtained from the Ethics Committee of Guilin Medical University.

### Clinical and biochemical characteristics

Clinical data of the subjects was collected using a standardized questionnaire and medical records, encompassing age, height, weight, systolic pressure (SBP), diastolic blood pressure (DBP), FPG, 1hPG, 2hPG, glycosylated hemoglobin (HbA1c), triglyceride (TG), total cholesterol (TC), high-density lipoprotein cholesterol (HDL-c), and low-density lipoprotein cholesterol (LDL-c), etc. Besides, the pre-pregnancy body mass index (pre-BMI) was calculated as weight (kg)/height (m)^2^.

### Genomic DNA extraction, variants selection, and genotyping

The genomic DNA was extracted from EDTA-treated peripheral whole blood using a DNA extraction kit (Aidlab Biotechnologies Co., Ltd., China) and stored at −80 °C. Candidate functional SNPs were identified based on our prior analysis using the Infinium Asian Screening Array (ASA) BeadChip, with selection criteria set at a significance level of *p* ≤ 5*10^−4^. Subsequently, SNP Function Prediction (FuncPred) tool^1^ was employed to screen for potential functional variants in the Chinese Han population in Beijing (CHB) with minimum allele frequencies exceeding 0.5.

The SNP was genotyped by the Sequenom MassARRAY platform. The PCR mix consisted of 1.0 μL of template DNA (20 ~ 100 ng/μL), 1.850 μL of ddH_2_O, 0.625 μL of 1.25 × PCR buffer (15 mmol/L MgCl_2_), 0.325 μL of 25 mmol/L MgCl_2_, 0.1 μL of 25 mmol/L dNTPs, 1 μL of 0.5 μmol/L primer mix, and 0.1 μL of 5 U/μL HotStar Taq polymerase. The PCR cycling conditions included an initial denaturation at 94 °C for 15 min, followed by 45 cycles of denaturation at 94 °C for 20 s, annealing at 56 °C for 30 s, extension at 72 °C for 1 min, and a final extension at 72 °C for 3 min. Finally, the original data and genotyping plots were generated using TYPER 4.0 software.

### Statistical analysis

The data analysis was conducted using IBM SPSS Statistics 28 for Windows (IBM Corp., Armonk, NY, United States) and R 4.3.1 software. Hardy–Weinberg equilibrium was detected by a χ^2^ goodness-of-fit test. The independent samples *t-tests* were employed to compare the distribution differences of clinical and biochemical variables between cases and controls, presented as the mean ± sd. The odds ratios (ORs) and their corresponding 95% confidence intervals (CIs) were calculated to evaluate the association between variants and GDM risk. Statistical significance was set at a two-sided *p* < 0.05. Stratified analysis was conducted to evaluate the association between positive SNP and GDM risk among different subgroups, categorized by the mean value of variables. Additionally, false-positive reporting probability (FPRP) analysis was utilized to address chance associations that could potentially lead to false-positive association findings.

Given the observed interaction between clinical factors (pre-BMI, DBP, FPG, and HbA1c) and genetic variants in the study, we hypothesized that they potentially influence the risk of GDM. Consequently, both univariate and multivariate MR analyses were performed. Exposure data were extracted from the IEU Open GWAS project^2^ with the following GWAS IDs: BMI (ukb-b-19953), DBP (ebi-a-GCST90018952), FPG (ebi-a-GCST90002232), and HbA1c (ieu-b-4842), while outcome data were obtained from FinnGen Consortium (GWAS ID: finngen_R10_GEST_DIABETES). Detailed information regarding the data sources is provided in Supplementary Table 1.

We identified independent single-nucleotide polymorphisms with low linkage disequilibrium (*r^2^* < 0.001) that showed significant associations with exposure factors (*p* < 5 × 10^−8^) and calculated the F-statistic using the equation F-statistic = beta^2^/se^2^ (19), where an F-statistic>10 indicated adequate instrument strength. The primary MR analysis was conducted using the inverse variance weighting (IVW) method. Additional sensitivity analyses were performed using the MR-Egger, weighted median, simple mode and weighted mode (25). Heterogeneity and pleiotropy were assessed by Cochran’s Q statistics and MR Pleiotropy RESidual Sum and Outlier (MR-PRESSO) methods. The directional validity of the causal relationships between exposure and outcome was assessed using the MR-Steiger test (26). Additionally, multivariate MR (MVMR) was conducted to assess whether confounding factors influenced the causal relationship between validaexposure and outcome. All statistical analyses were carried out using R (v4.3.1) with the R packages “TwoSampleMR,” “MR-PRESSOR,” and “MendelianRandomization.” A detailed flowchart illustrating this complete MR analysis pipeline has been provided as Supplementary Figure 2.

A predictive nomogram integrating clinical risk factors and positive SNPs was developed to assess the risk of GDM. Scores corresponding to each risk factor were aggregated to derive a total score, facilitating risk evaluation. The subjects were randomly divided into training and validation sets at a ratio of 7:3. Receiver operating characteristic (ROC) curves and calibration plots were generated, and sensitivity and specificity were calculated to evaluate the predictive ability of the nomogram. Meanwhile, decision curve analysis (DCA) was performed to evaluate the clinical utility and benefit of the nomogram. The analyses were conducted using R packages (v4.3.3).

## Results

### Patient characteristics

The characteristics of the subjects are presented in Table 1. In cases of GDM, the age, pre-BMI, SBP, DBP, FPG, 1hPG, 2hPG, HbAlc, TG were significantly higher compared to those in healthy pregnancies (*p* < 0.001).

**Table 1:** Demographic and clinical characteristics in cases and controls.

Variables	GDM (*n* = 554)	Controls (*n* = 640)	*t*	*P*
Age (years)	31.55 ± 4.76	28.83 ± 4.13	10.44	<0.001
Pre-pregnancy BMI (Kg/m^2^)	23.14 ± 3.61	21.44 ± 3.00	8.72	<0.001
SBP (mmHg)	111.61 ± 10.59	108.76 ± 9.38	4.89	<0.001
DBP (mmHg)	70.51 ± 8.74	68.68 ± 7.90	3.78	<0.001
FPG (mmol/L)	5.22 ± 1.33	4.41 ± 0.37	13.96	<0.001
1hPG (mmol/L)	9.76 ± 2.25	6.96 ± 1.43	25.3	<0.001
2hPG (mmol/L)	8.30 ± 2.17	6.08 ± 1.10	21.85	<0.001
HbA1c (%)	5.44 ± 0.68	5.00 ± 0.48	12.62	<0.001
TG (mmol/L)	2.67 ± 1.20	2.43 ± 1.01	3.82	<0.001
TC (mmol/L)	5.37 ± 1.15	5.29 ± 1.07	1.3	0.194
HDL-c (mmol/L)	1.66 ± 0.42	1.65 ± 0.40	0.31	0.756
LDL-c (mmol/L)	3.49 ± 1.02	3.45 ± 1.01	0.68	0.496

SBP, systolic blood pressure; DBP, diastolic blood pressure; FPG, fasting plasma glucose; 1hPG, 1-h plasma glucose; 2hPG, 2-h plasma glucose; HbA1c, Hemoglobin A1c; TG, Triglyceride; TC, Total cholesterol; HDL-c, High-Density Lipoprotein cholesterol; LDL-c, Low-Density Lipoprotein Cholesterol.

### Genotype distribution of the studied variants

The frequency distribution of rs6127416 followed the Hardy Weinberg equilibrium law (*P*_HWE_ > 0.05). Significantly different genotype distributions of rs6127416 were observed between GDM patients and controls (*p* < 0.001). Following adjustment for age and pre-pregnancy BMI, rs6127416 exhibited a significant association with the risk of GDM. Compared to the TT genotype, rs6127416 AA showed a substantial increase in the risk of GDM (adjusted OR = 2.20, 95%CI = 1.53–3.18, *p* < 0.001). In the recessive model, in comparison to TT/TA genotypes, the AA genotype also demonstrated a pronounced effect on the susceptibility to GDM (adjusted OR = 2.35, 95%CI = 1.68–3.30, *p* < 0.001). These findings are summarized in Table 2.

**Table 2:** Positive association analysis of genetic variants with GDM risk.

Genotype	Case	Control	*P* ^a^	Crude OR (95%CI)	*P* ^b^	Adjusted OR (95%CI)	*P* ^c^
rs6127416							
TT	200	256	<0.001	1		1	
TA	231	312		0.95 (0.74–1.22)	0.675	0.89 (0.68–1.16)	0.377
AA	123	73		2.16 (1.53–3.04)	<0.001	2.20 (1.53–3.18)	<0.001
TA/AA	354	385		1.18 (0.93–1.49)	0.174	1.13 (0.88–1.45)	0.343
TT/TA	431	568		1		1	
AA	123	73		2.22 (1.62–3.04)	<0.001	2.35 (1.68–3.30)	<0.001

^a^Genotype distribution difference tested by c^2^. ^b^Unconditional logistic regression analysis. ^c^Adjusted for age, pre-BMI in logistic regression models. *P* < 0.05 was statistical significance.

Stratified analysis was employed to investigate the association between rs6127416 and GDM risk using the recessive model. The AA genotype of rs6127416 was found to significantly elevate the risk of GDM in all subgroups, with the exception of individuals with FPG levels >4.79 mmol/L. Interestingly, notable interactive effects were observed between the genetic loci rs6127416 and pre-BMI (*P*_interaction_ = 0.022), DBP (*P*_interaction_ = 0.039), FPG (*P*_interaction_ < 0.001), and HbA1c (*P*_interaction_ < 0.001) (Table 3).

**Table 3:** Stratification analysis for associations between rs6127416 and GDM risk.

Variables	AA (case/control)	TT/TA (case/control)	Crude OR (95%CI)	*P* ^a^	Adjusted OR (95%CI)	*P* ^b^	*P* ^c^
Age (year)							0.395
≤30.09	55/52	194/397	2.16 (1.43–3.28)	0.000	2.36 (1.52–3.65)	0.000	
>30.09	68/21	237/170	2.32 (1.37–3.94)	0.002	2.36 (1.38–4.05)	0.002	
Pre-BMI (Kg/m^2^)							0.022
≤22.23	62/56	181/377	2.31 (1.54–3.45)	0.000	2.22 (1.46–3.39)	0.000	
>22.23	61/17	249/189	2.72 (1.54–4.82)	0.001	2.74 (1.53–4.92)	0.001	
SBP (mmHg)							0.490
≤110.08	56/44	210/328	1.99 (1.29–3.06)	0.002	1.94 (1.23–3.07)	0.005	
>110.08	67/29	221/239	2.50 (1.56–4.01)	0.000	2.92 (1.76–4.85)	0.000	
DBP (mmHg)							0.039
≤69.53	57/36	214/306	2.26 (1.44–3.56)	0.000	2.26 (1.41–3.64)	0.001	
>69.53	66/37	217/261	2.15 (1.38–3.33)	0.001	2.51 (1.55–4.05)	0.000	
FPG (mmol/L)							<0.001
≤4.79	55/62	161/503	2.77 (1.85–4.15)	0.000	2.99 (1.94–4.59)	0.000	
>4.79	68/11	270/64	1.47 (0.73–2.93)	0.280	1.48 (0.72–3.03)	0.289	
1h-PG (mmol/L)							0.099
≤8.26	26/58	102/449	1.97 (1.19–3.29)	0.009	1.84 (1.08–3.13)	0.026	
>8.26	97/15	329/118	2.32 (1.30–4.16)	0.005	2.74 (1.50–5.00)	0.001	
2h-PG (mmol/L)							0.497
≤7.11	36/61	120/469	2.31 (1.46–3.65)	0.000	2.18 (1.34–3.54)	0.002	
>7.11	87/12	311/98	2.29 (1.20–4.35)	0.012	2.73 (1.41–5.31)	0.003	
HbA1c (%)							<0.001
≤5.20	52/59	148/416	2.48 (1.63–3.76)	0.000	2.42 (1.57–3.72)	0.000	
>5.20	71/14	283/151	2.71 (1.48–4.96)	0.001	3.05 (1.62–5.75)	0.001	
TG (mmol/L)							0.159
≤2.54	68/47	221/354	2.32 (1.54–3.49)	0.000	2.21 (1.43–3.43)	0.000	
>2.54	55/26	210/213	2.15 (1.30–3.55)	0.003	2.60 (1.52–4.44)	0.000	

^a^Unconditional logistic regression analysis, ^b^adjusted for age, pre-BMI in logistic regression models, ^c^Test for multiplicative interaction obtained from logistic regression models.

### FPRP analysis

The false-positive reporting probability (FPRP) analysis was employed to validate the robustness of the statistically significant associations between variants and GDM risk, employing a predefined FPRP cutoff value of 0.2 and a prior probability level of 0.1. Associations with FPRP values <0.2 were considered genuine. Subsequently analysis indicated that the association between rs6127416 and GDM risk in subjects with AA genotype compared to TT, AA compared to TT/TA, age ≤ 30.09 years, pre-BMI ≤ 22.23 Kg/m^2^, SBP > 110.08 mmHg, DBP, FPG ≤ 4.79 mmol/L, 2hPG ≤ 7.11 mmol/L, HbAlc ≤ 5.20%, and TG appeared to be genuine. Other positive associations observed should be interpreted with caution, as they may have been obtained by chance, as detailed in Table 4.

**Table 4:** FPRP analysis of the significant associations and GDM risk.

Comparisons	Adjusted OR (95%CI)	Adjusted *p*-value	Prior probability					
			0.25	0.1	0.01	0.001	0.0001	0.00001
rs6127416								
AA vs. TT	2.20 (1.53–3.18)	0.000024	0.004	0.011	0.109	0.553	0.925	0.992
AA vs. TT/TA	2.35 (1.68–3.30)	0.000001	0.001	0.004	0.040	0.294	0.806	0.977
Subgroup								
Age (years)								
≤30.09	2.36 (1.52–3.65)	0.000117	0.016	0.047	0.353	0.847	0.982	0.998
>30.09	2.36 (1.38–4.05)	0.002000	0.102	0.254	0.790	0.974	0.997	1.000
pre-BMI (Kg/m^2^)								
≤22.23	2.22 (1.46–3.39)	0.000209	0.018	0.053	0.381	0.861	0.984	0.998
>22.23	2.74 (1.53–4.92)	0.001000	0.101	0.253	0.788	0.974	0.997	1.000
SBP (mmHg)								
≤110.08	1.94 (1.23–3.07)	0.005000	0.096	0.242	0.778	0.973	0.997	1.000
>110.08	2.92 (1.76–4.85)	0.000034	0.020	0.058	0.404	0.873	0.986	0.999
DBP (mmHg)								
≤69.53	2.26 (1.41–3.64)	0.001000	0.054	0.147	0.654	0.950	0.995	0.999
>69.53	2.51 (1.55–4.05)	0.000181	0.028	0.080	0.490	0.907	0.990	0.999
FPG (mmol/L)								
≤4.79	2.99 (1.94–4.59)	0.000001	0.002	0.006	0.065	0.413	0.875	0.986
1hPG (mmol/L)								
≤8.26	1.84 (1.08–3.13)	0.026000	0.251	0.502	0.917	0.991	0.999	1.000
>8.26	2.74 (1.50–5.00)	0.001000	0.109	0.269	0.802	0.976	0.998	1.000
2hPG (mmol/L)								
≤7.11	2.18 (1.34–3.54)	0.002000	0.076	0.197	0.730	0.965	0.996	1.000
>7.11	2.73 (1.41–5.31)	0.003000	0.191	0.415	0.886	0.987	0.999	1.000
HbA1c (%)								
≤5.20	2.42 (1.57–3.72)	0.000062	0.012	0.035	0.283	0.800	0.976	0.998
>5.20	3.05 (1.62–5.75)	0.001000	0.126	0.302	0.826	0.980	0.998	1.000
TG (mmol/L)								
≤2.54	2.21 (1.43–3.43)	0.000383	0.028	0.078	0.483	0.904	0.990	0.999
>2.54	2.60 (1.52–4.44)	0.000457	0.059	0.159	0.676	0.955	0.995	1.000

### Causal effect of clinical indicators on GDM

Based on the established screening criteria, a total of 428 SNPs predicting BMI, 186 SNPs predicting DBP, 55 SNPs predicting FPG, and 30 SNPs predicting HbA1c were included for MR analysis in this study (Table 5). Each set of SNPs associated with the respective exposure variables exhibited adequate instrument strength, with F-statistics exceeding 10.

**Table 5:** The significant causal associations of clinical indicators with GDM.

Outcome	Exposure	Method	Number of SNPs	β	SE	OR (95%CI)	*P*-value	Heterogeneity test				Pleiotropy test
								MR egger		IVW		MR-PRESSO
								Q	*P* value	Q	*P*_value_	*P*_Global Test_
GDM	BMI	IVW	428	0.561	0.047	1.75 (1.60–1.92)	<0.001	636.70	<0.001	638.68	<0.001	0.250
		MR egger	428	0.701	0.131	2.02 (1.56–2.61)	<0.001					
		Weighted median	428	0.672	0.071	1.96 (1.70–2.25)	<0.001					
		Simple mode	428	0.622	0.246	1.86 (1.15–3.02)	0.012					
		Weighted mode	428	0.728	0.132	2.07 (1.60–2.68)	<0.001					
	FPG	IVW	55	1.593	0.149	4.92 (3.67–6.59)	<0.001	124.31	<0.001	126.55	<0.001	0.332
		MR egger	55	1.347	0.293	3.85 (2.17–6.82)	<0.001					
		Weighted median	55	1.355	0.204	3.88 (2.60–5.79)	<0.001					
		Simple mode	55	2.339	0.520	10.37 (3.75–28.71)	<0.001					
		Weighted mode	55	1.117	0.193	3.05 (2.09–4.46)	<0.001					
	HbA1c	IVW	30	0.261	0.081	1.30 (1.11–1.52)	0.001	121.62	<0.001	124.66	<0.001	0.410
		MR egger	30	0.132	0.174	1.14 (0.81–1.61)	0.457					
		Weighted median	30	0.029	0.069	1.03 (0.90–1.18)	0.670					
		Simple mode	30	−0.022	0.100	0.98 (0.80–1.19)	0.827					
		Weighted mode	30	−0.022	0.072	0.98 (0.85–1.13)	0.762					
	DBP	IVW	186	0.024	0.070	1.02 (0.89–1.18)	0.730	259.62	<0.001	264.47	<0.001	0.065
		MR egger	186	−0.362	0.220	0.70 (0.45–1.07)	0.101					
		Weighted median	186	0.029	0.096	1.03 (0.85–1.24)	0.763					
		Simple mode	186	0.118	0.296	1.13 (0.63–2.01)	0.690					
		Weighted mode	186	0.044	0.274	1.04 (0.61–1.79)	0.873					

IVW, inverse variance weighting; MR-PRESSO, MR Pleiotropy RESidual Sum and Outlier.

**Figure 1:**
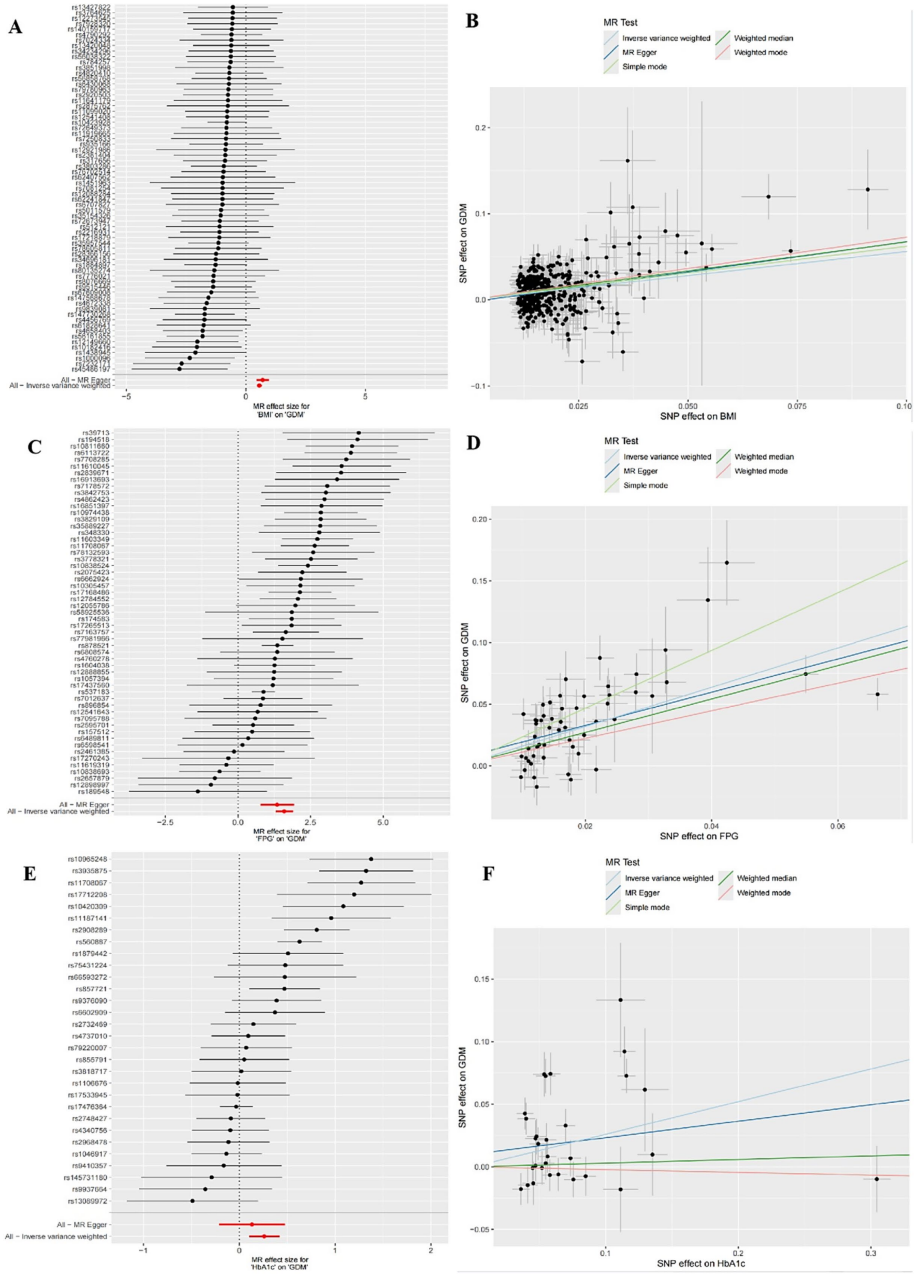
Forest plots of univariate mendelian randomization (UVMR) results and scatter plots estimating the causal effect of clinical indicators on GDM. **(A,B)** Forest plot and scatter plot for the causal effect of BMI. **(C,D)** Forest plot and scatter plot for the causal effect of FPG. **(E,F)** Forest plot and scatter plot for the causal effect of HbA1c.

**Figure 2:**
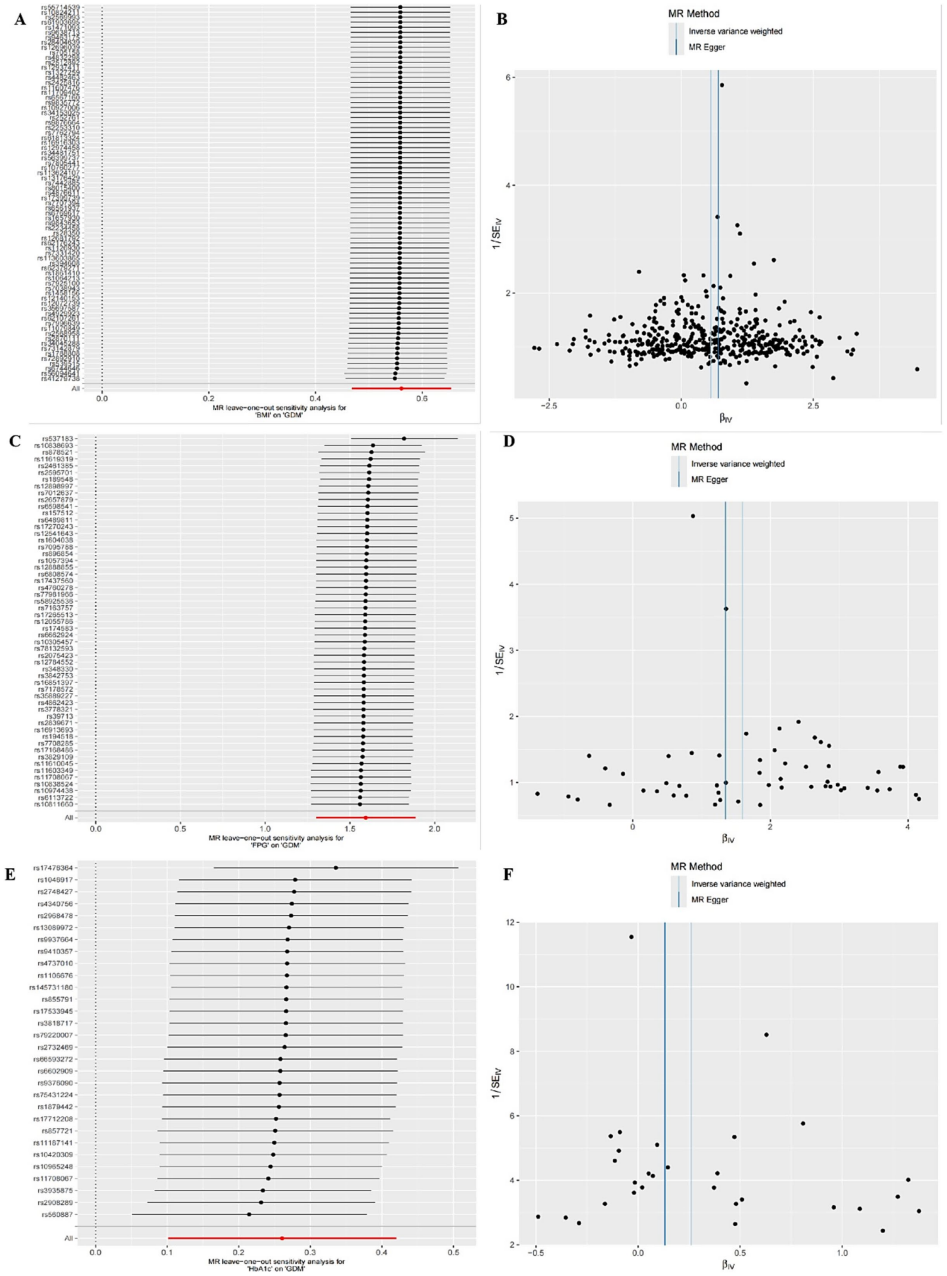
Sensitivity analysis for UVMR through leave-one-out analysis and funnel plot. **(A,B)** The leave-one-out plot and funnel plot of MR estimates for BMI associated with GDM. **(C,D)** The leave-one-out plot and funnel plot of MR estimates for FPG associated with GDM. **(E,F)** The leave-one-out plot and funnel plot of MR estimates for HbA1c associated with GDM.

The IVW random effects model was employed as the primary method for MR analysis due to the detection of heterogeneity in the study through Cochran’s Q statistics (*P*_heterogeneity_ < 0.05). The results showed a significant causal association between BMI, FPG and HbA1c with GDM (ORBMI = 1.75, 95% CI: 1.60–1.92, *p* < 0.001; ORFPG = 4.92, 95% CI: 3.67–6.59, *p* < 0.001; ORHbA1c = 1.30, 95% CI: 1.11–1.52, *p* = 0.001) (Table 5 and Figures 1A–F). Reciprocal MR analyses, including MR-Egger, weighted median, simple mode and weighted mode, consistently supported the direction of effect in the causal estimation of BMI and FPG, and GDM. The MR-PRESSO global test indicated no evidence of horizontal pleiotropy in the identified associations (*p* > 0.05) (Table 5). Symmetrical funnel plots suggested the reliability of the MR analyses (Figures 2B,D,F). Leave-one-out analysis indicated that the observed causal findings were not influenced by any single SNP (Figures 2A,C,E). Results from the MR-Steiger directionality test demonstrated that the correctness of the directions of causal inference (Table 6). However, there was no evidence of causal associations between DBP and GDM (Table 5).

**Table 6:** MR Steiger directionality test for causal direction.

Exposure	Outcome	snp_r^2^.exposure	snp_r^2^.outcome	Causal direction	*P*_value_
BMI	GDM	0.058	2.8 × 10–3	TRUE	<0.001
FPG	GDM	0.026	1.30 × 10–3	TRUE	<0.001
HbA1c	GDM	0.069	5.58 × 10–4	TRUE	<0.001
FPG	GDM	0.030	8.73 × 10–4	TRUE	<0.001

The variance in the outcome (snp_r^2^.outcome) was less than each exposure (snp_r^2^.exposure), indicating the causal directions that exposures cause outcome were correct.

**Figure 3:**
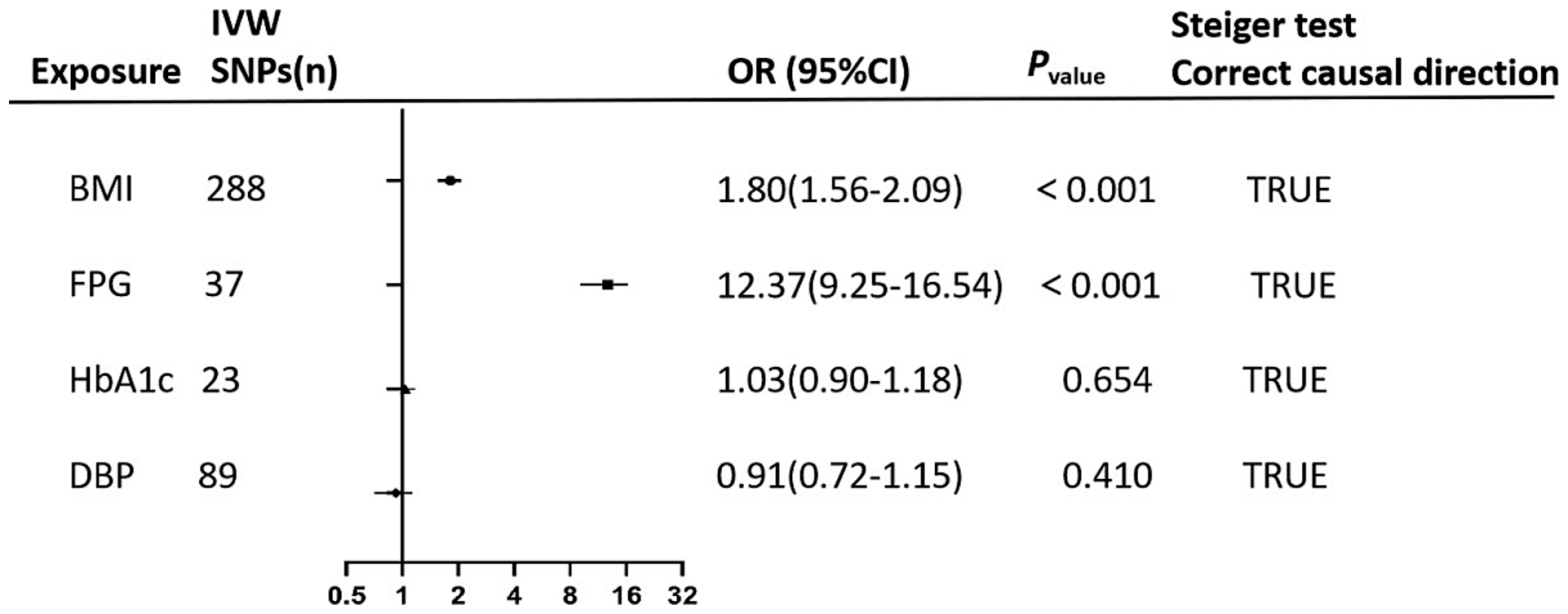
The forest plot performs the findings of the multivariate mendelian randomization (MVMR) analyses on the causal effects of clinical indicators (BMI, FPG, HbA1c, and DBP) on GDM using IVW method. Steiger test confirms the correctness of the causal directions indicating that exposure leads to the outcome.

In multivariable MR analysis, IVW method indicated persistent positive causal associations of BMI (OR_BMI_ = 1.80, 95%CI: 1.56–2.09, *p* < 0.001), and FPG (OR_FPG_ = 12.37, 95% CI: 9.25–16.54, *p* < 0.001) with GDM risk. However, the causal association of HbA1c with GDM was no longer statistically significant after adjusting for BMI, FPG, and DBP (Figure 3).

### Nomogram model construction

**Figure 4:**
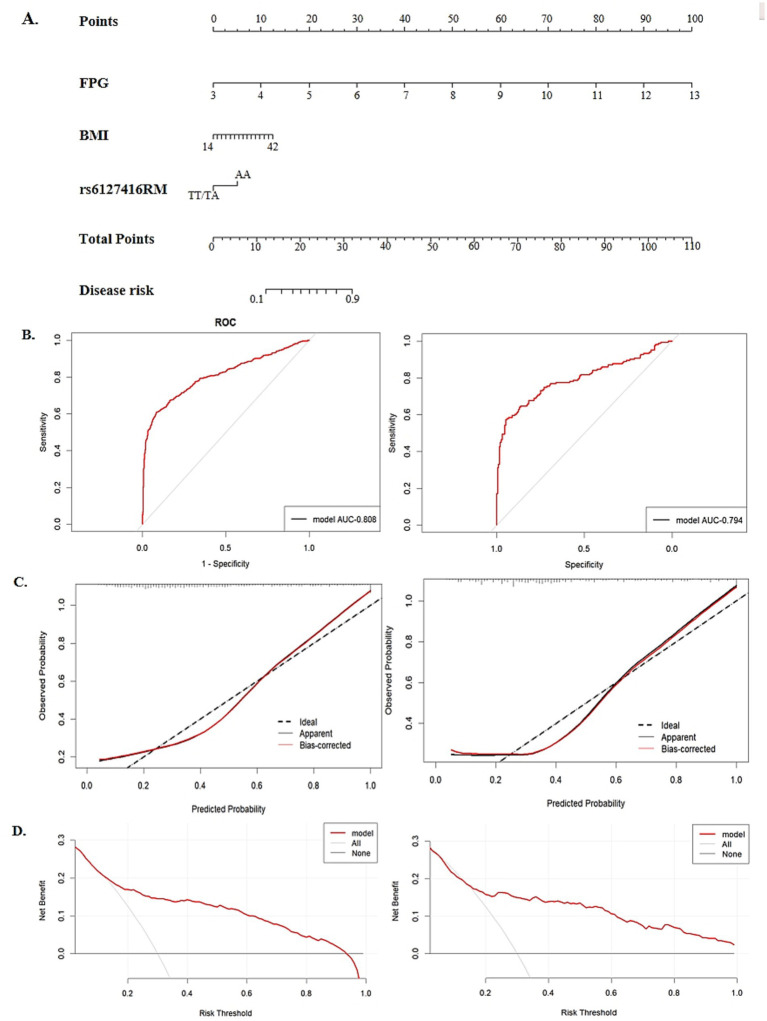
The establishment and validation of a nomogram model in predicting GDM risk. **(A)** A nomogram model for predicting the risk of GDM, constructed by pre-BMI, FPG, rs6127416. **(B)** The area under the receiver operating characteristic curve (ROC) in the training group and validation group. **(C)** Calibration plot of nomogram in the training group and validation group. **(D)** The decision curve analysis (DCA) in the training group and validation group.

Finally, three risk factors were identified for nomogram construction, including pre-BMI, FPG, and the recessive model of rs6127416, as illustrated in Figure 4A. The area under the ROC curve (AUC) was 0.808 in the training set and 0.794 in the testing set, indicating a strong predictive and discriminatory performance (Figure 4B). Calibration curves displayed minimal deviation between nomogram predictions and actual observations in both the training and testing sets, confirming a good fit of the model (Figure 4C). DCA revealed that the model curves for various risk threshold probabilities surpassed the reference lines (“treat all” or “treat none”), indicating a favorable net clinical benefit of the nomogram model in both the training and testing sets (Figure 4D).

## Discussion

GDM represents one of the most prevalent complications during pregnancy, drawing substantial attention due to its detrimental impact on the health of both mothers and offspring in the short and long term. Despite significant advancements in understanding the susceptibility mechanisms of GDM, a comprehensive comprehension remains elusive. Recent investigations utilizing candidate gene studies and GWAS have identified numerous potential risk SNPs, highlighting the genetic predisposition associated with GDM (6, 27). The development of GDM is affected by a complex interplay of genetic variants, diverse environmental exposures, and their interactions. In this study, we explored the genetic and environmental causal factors related to GDM and successfully constructed an individualized GDM risk prediction model. This endeavor offers valuable insights for the identification and early intervention of high-risk populations susceptible to GDM (28).

This study provides evidence supporting the strong association between the genetic variant rs6127416 and the risk of GDM in a southern Chinese population, emphasizing the role of SNPs in the genetic predisposition to GDM. Furthermore, stratified analysis revealed varying effects of the SNP rs6127416 on clinical parameters across different subgroups. Significant interactions were also observed between SNPs and multiple clinical variables, such as pre-BMI, DBP and HbA1c. Additionally, Huang X *et al*’s study also suggested that genetic variants (LINGO2 rs10968576, rs1412239, GLIS3 rs10814916) could potentially diminish the protective properties of O_3_ against anti-inflammatory responses, which may confer some level of protection against GDM (29). These results collectively highlight the combined impact of genetic variants, environmental factors, and clinical indicators on individual susceptibility to GDM, indicating potential modifying or interactive effects.

FPRP analysis suggested that the association detected between rs6127416 in recessive genetic model and GDM risk within certain subgroups is robust and reliable. However, FPRP values in other subgroups exceeded 0.2, suggesting that these associations may have been obtained by chance and should be interpreted cautiously.

The MR methods confirmed the causal relationship between clinical indicators (pre-BMI, FPG) and GDM. Due to the unknown biological function of genetic variant, we cannot entirely eliminate the potential influence of horizontal pleiotropy. To ensure the accuracy of this causal inference, we evaluated potential pleiotropy using MR-PRESSO, which revealed no evidence of horizontal pleiotropy (*p* > 0.05). Results underscore the role of lipid metabolism disturbances in glucose homeostasis. Obesity contributes to heightened plasma free fatty acid (FFA) and elevated triglycerides, leading to intracellular lipid accumulation in non-adipose cells (30). Ectopic lipid accumulation in these cells can induce insulin resistance through inflammatory mediators and oxidative stress pathways (31, 32). Ultimately, this cascade can lead to disruptions in glucose metabolism. Therefore, preventive measures targeting pre-pregnancy obesity are crucial in mitigating the incidence of GDM.

Taken together, our case–control and MR analyses collectively demonstrate that pre-pregnancy adiposity and elevated FPG are causal risk factors for GDM. Results also suggest causal factors of GDM are modified through lifestyle interventions. Additionally, Our MR analyses revealed that the significant association between HbA1c and GDM observed in univariate analysis did not persist in multivariable models. This suggests that HbA1c is likely to serve as a biomarker of underlying dysglycemia rather than an independent causal factor in GDM development.

A nomogram represents a mathematical model that visually and comprehensively assesses the risk of a disease, offering a practical approach to estimating the likelihood of clinical events. Essentially, the construction of a predictive model acts as a crucial link between clinical epidemiology or molecular epidemiology and clinical practice (33, 34). In this study, validated SNPs significantly associated with GDM and clinical indicators (pre-BMI, FPG) were integrated to construct a nomogram model for predicting GDM risk. The calibration curve demonstrated a close fit to the ideal curve, indicating good model accuracy. The ROC curve illustrated the strong predictive capability of this model, while DCA highlighted its promising clinical utility. The developed nomogram offers a practical tool for early-risk stratification, potentially integrated into first-trimester care via digital calculators or clinical sheets. Focusing on women at highest predicted risk (top 20%) may facilitate early intervention and preventive efforts.

However, this study still has certain limitations that warrant consideration. Firstly, while certain potential confounding factors were accounted for, the absence of data regarding diet, physical activity, smoking status, parity, poor obstetrics, malnutrition, and socioeconomic factors, and other relevant variables may introduce bias and affect the accuracy of the associations between genetic variants and GDM risk. Secondly, being a hospital-based case–control study, inherent selection bias in subject recruitment and data collection processes cannot be overlooked. Thirdly, despite the relatively large sample size, the limited frequency of genotypes assessed in the studied variants may restrict the statistical robustness of the analysis. Finally, the validation cohort was obtained from a random split of the original cohort, which may potentially affect the independence and reliability of the validation results. Independent external validation should be incorporated in future studies to better confirm the model and improve its practical value.

In conclusion, this study confirms the substantial impact of genetic and environmental factors on the development of GDM. The clinical indicators, particularly pre-BMI and FPG, exhibit significant positive causal effects on GDM. The integration of key genetic SNPs and clinical indicators, such as rs6127416, pre-BMI, FPG, in a predictive model enables the effective differentiation of individual GDM risk during early pregnancy.

## Data Availability

The genotyping and phenotypic data are publicly available in the Dryad repository at https://doi.org/10.5061/dryad.gmsbcc322.

## Footnotes

^1^ http://manticore.niehs.nih.gov/snpinfo/snpfunc.html

^2^ https://gwas.mrcieu.ac.uk/

## References

[1] Sweeting A Wong J Murphy HR Ross GP. A clinical update on gestational diabetes mellitus. Endocr Rev. (2022) 43:763–93. doi: 10.1210/endrev/bnac003, PMID: 35041752 PMC9512153

[2] Gao C Sun X Lu L Liu F Yuan J. Prevalence of gestational diabetes mellitus in mainland China: a systematic review and Meta-analysis. J Diabetes Investig. (2019) 10:154–62. Epub 2018/04/24. doi: 10.1111/jdi.12854, PMID: 29683557 PMC6319492

[3] Farahvar S Walfisch A Sheiner E. Gestational diabetes risk factors and long-term consequences for both mother and offspring: a literature review. Exp Rev Endocrinol Metabol. (2018) 14:63–74. doi: 10.1080/17446651.2018.1476135 30063409

[4] Li M Rahman ML Wu J Ding M Chavarro JE Lin Y. Genetic factors and risk of type 2 diabetes among women with a history of gestational diabetes: findings from two independent populations. BMJ Open Diabetes Res Care. (2020) 8:e000850. Epub 2020/01/21. doi: 10.1136/bmjdrc-2019-000850 PMC7039588 31958311

[5] Chukwuemeka S Chivese T Gopinath A Obikeze K. Adverse pregnancy outcomes in gestational diabetes mellitus: a systematic review and Meta-analysis protocol. BMJ Open. (2024) 14:e058625. doi: 10.1136/bmjopen-2021-058625, PMID: 38803262 PMC11328635

[6] Plows JF Stanley JL Baker PN Reynolds CM Vickers MH. The pathophysiology of gestational diabetes mellitus. Int J Mol Sci. (2018) 19:3342. doi: 10.3390/ijms19113342 30373146 PMC6274679

[7] You H Hu J Liu Y Luo B Lei A. Risk of type 2 diabetes mellitus after gestational diabetes mellitus: a Systematic Review & Meta-Analysis. Indian J Med Res. (2021) 154:62–77. Epub 2021/11/17. doi: 10.4103/ijmr.IJMR_852_18, PMID: 34782531 PMC8715678

[8] Chen P Wang S Ji J Ge A Chen C Zhu Y. Risk factors and Management of Gestational Diabetes. Cell Biochem Biophys. (2015) 71:689–94. Epub 2014/10/02. doi: 10.1007/s12013-014-0248-2, PMID: 25269773

[9] Monod C Kotzaeridi G Linder T Eppel D Rosicky I Filippi V. Prevalence of gestational diabetes mellitus in women with a family history of type 2 diabetes in first- and second-degree relatives. Acta Diabetol. (2022) 60:345–51. doi: 10.1007/s00592-022-02011-w, PMID: 36508047 PMC9931850

[10] Zhou Y Xie N Zhang L Chen D. Impact of family history of diabetes on blood glucose, lipid levels and perinatal outcomes in pregnant women with gestational diabetes mellitus. J Zhejiang Univ. (2021) 50:329–34. doi: 10.3724/zdxbyxb-2021-0193, PMID: 34402261 PMC8710266

[11] Chaudhary R Singh B Kumar M Gakhar SK Saini AK Parmar VS. Role of single nucleotide polymorphisms in pharmacogenomics and their association with human diseases. Drug Metab Rev. (2015) 47:281–90. Epub 2015/05/23. doi: 10.3109/03602532.2015.1047027, PMID: 25996670

[12] Prasad RB Kristensen K Katsarou A Shaat N. Association of Single Nucleotide Polymorphisms with insulin secretion, insulin sensitivity, and diabetes in women with a history of gestational diabetes mellitus. BMC Med Genet. (2021) 14:274. doi: 10.1186/s12920-021-01123-6, PMID: 34801028 PMC8606068

[13] Robert F Pelletier J. Exploring the impact of single-nucleotide polymorphisms on translation. Front Genet. (2018) 9:9. doi: 10.3389/fgene.2018.00507, PMID: 30425729 PMC6218417

[14] Uffelmann E Huang QQ Munung NS de Vries J Okada Y Martin AR. Genome-wide association studies. Nat Rev Methods Primers. (2021) 1:59. doi: 10.1038/s43586-021-00056-9

[15] Huang G Liang Q Wang Y Qin L Yang H Lin L. Association of Ace2 gene functional variants with gestational diabetes mellitus risk in a southern Chinese population. Front Endocrinol. (2022):13. doi: 10.3389/fendo.2022.1052906 PMC9752565 36531495

[16] Li R Wang Y Yang L Zhong P Huang G Liang Q. Genetic variants of Erbb4 gene and Risk of gestational diabetes mellitus: a susceptibility and diagnostic nomogram study. Front Endocrinol. (2023) 14:1283539. Epub 2023/12/27. doi: 10.3389/fendo.2023.1283539, PMID: 38149095 PMC10749950

[17] Liang Q Li M Huang G Li R Qin L Zhong P. Genetic susceptibility, Mendelian randomization, and nomogram model construction of gestational diabetes mellitus. J Clin Endocrinol Metabol. (2024) 109:2802–14. doi: 10.1210/clinem/dgae200 38625888

[18] Yu X-y Song L-p Zheng H-t Wei S-d Wen X-l Huang B. Association between functional genetic variants in retinoid X receptor-Α/Γand the risk of gestational diabetes mellitus in a southern Chinese population. Biosci Rep. (2021) 41:BSR20211338. doi: 10.1042/bsr20211338, PMID: 34633445 PMC8529336

[19] Lawlor DA Harbord RM Sterne JA Timpson N Davey Smith G. Mendelian randomization: using genes as instruments for making causal inferences in epidemiology. Stat Med. (2008) 27:1133–63. doi: 10.1002/sim.3034, PMID: 17886233

[20] Nattel S. Canadian journal of cardiology January 2013: genetics and more. Can J Cardiol. (2013) 29:1–2. Epub 2012/12/25. doi: 10.1016/j.cjca.2012.11.015, PMID: 23261318

[21] Li W Li Y Zhang Z Xia K Shang X Yang X. Predictive nomogram of rage genetic polymorphisms and metabolic risk factors for myocardial infarction risk in a Han Chinese population. Angiology. (2017) 68:877–83. doi: 10.1177/0003319717696622, PMID: 28956473

[22] Tong JN Wu LL Chen YX Guan XN Tian FY Zhang HF. Fasting plasma glucose in the first trimester is related to gestational diabetes mellitus and adverse pregnancy outcomes. Endocrine. (2022) 75:70–81. Epub 2021/08/04. doi: 10.1007/s12020-021-02831-w, PMID: 34342804 PMC8763802

[23] Li S Li H Li C He X Wang Y. Development and validation of a nomogram for predicting the risk of pregnancy-induced hypertension: a retrospective cohort study. J Womens Health. (2021) 30:1182–91. Epub 2020/10/31. doi: 10.1089/jwh.2020.8575 33121332

[24] Metzger BE Gabbe SG Persson B Buchanan TA Catalano PA Damm P. International association of diabetes and pregnancy study groups recommendations on the diagnosis and classification of hyperglycemia in pregnancy. Diabetes Care. (2010) 33:676–82. Epub 2010/03/02. doi: 10.2337/dc09-1848 20190296 PMC2827530

[25] Slob EAW Burgess S. A comparison of robust Mendelian randomization methods using summary data. Genet Epidemiol. (2020) 44:313–29. Epub 2020/04/07. doi: 10.1002/gepi.22295, PMID: 32249995 PMC7317850

[26] Hemani G Tilling K Davey Smith G. Orienting the causal relationship between imprecisely measured traits using GWAS summary data. PLoS Genet. (2017) 13:e1007081. doi: 10.1371/journal.pgen.1007081, PMID: 29149188 PMC5711033

[27] Xie K Zhang Y Wen J Chen T Kong J Zhang J. Genetic predisposition to gestational glucose metabolism and gestational diabetes mellitus risk in a Chinese population. J Diabetes. (2019) 11:869–77. doi: 10.1111/1753-0407.12923 30912250

[28] Zhang D Zhang S Li G Lai Y Yt H Wq C. A clinical model and nomogram for early prediction of gestational diabetes based on common maternal demographics and routine clinical parameters. J Obstet Gynaecol Res. (2022) 48:2738–47. doi: 10.1111/jog.15380 35909297

[29] Huang X Liang W Yang R Jin L Zhao K Chen J. Variations in the Lingo2 and Glis3 genes and gene-environment interactions increase gestational diabetes mellitus risk in Chinese women. Environ Sci Technol. (2024) 58:11596–605. Epub 2024/06/18. doi: 10.1021/acs.est.4c03221, PMID: 38888423

[30] Xu Q Zhou Q Yang Y Liu F Wang L Wang Q. Maternal pre-conception body mass index and fasting plasma glucose with the risk of pre-term birth: a cohort study including 4.9 million Chinese women. Front Reprod Health. (2021) 3:622346. doi: 10.3389/frph.2021.622346 36304061 PMC9580732

[31] Spradley FT Yen IW Lee C-N Lin M-W Fan K-C Wei J-N. Overweight and obesity are associated with clustering of metabolic risk factors in early pregnancy and the risk of Gdm. PLoS One. (2019) 14:e0225978. doi: 10.1371/journal.pone.0225978 31794594 PMC6890240

[32] Tanaka MJEJ. Molecular mechanism of obesity-induced adipose tissue inflammation; the role of Mincle in adipose tissue fibrosis and ectopic lipid accumulation. Endocr J. (2020) 67:107–11. doi: 10.1507/endocrj.EJ19-0417 31852849

[33] Lin Q Fang ZJ. Establishment and evaluation of a risk prediction model for gestational diabetes mellitus. World J Diabetes. (2023) 14:1541–50. Epub 2023/11/16. doi: 10.4239/wjd.v14.i10.1541, PMID: 37970129 PMC10642414

[34] Niu ZR Bai LW Lu Q. Establishment of gestational diabetes risk prediction model and clinical verification. J Endocrinol Investig. (2024) 47:1281–7. Epub 2023/12/12. doi: 10.1007/s40618-023-02249-3, PMID: 38085430 PMC11035389

