# Supplementary material for: Causal inference and risk prediction of gestational diabetes mellitus based on case–control study and Mendel randomization
Source: Front Nutr. 2025 Nov 3;12:1665813. doi: 10.3389/fnut.2025.1665813 (PMC12620502; doi:10.3389/fnut.2025.1665813)
Supplement: Supplementary file 1 [file Table_1.docx]

| Supplement Table 1. Characteristics of the summary GWAS data | | | | | | |
| --- | --- | --- | --- | --- | --- | --- |
| Category | Phenotype | Population | Year | Variable  type | Sample size | GWAS-ID |
| Outcomes |  |  |  |  |  |  |
|  | GDM | FinnGen | 2022 | Binary | 303,100 | finngen_R10_GEST_DIABETES |
| Exposures |  |  |  |  |  |  |
|  | BMI | European | 2018 | Continuous | 461,460 | ukb-b-19953 |
|  | HbA1c | European | 2022 | Continuous | 45,734 | ieu-b-4842 |
|  | FPG | European | 2021 | Continuous | 200,622 | ebi-a-GCST90002232 |
|  | DBP | European | 2021 | Continuous | 340,162 | ebi-a-GCST90018952 |
