# Supplementary figures and images for: Causal inference and risk prediction of gestational diabetes mellitus based on case–control study and Mendel randomization
Source: Front Nutr. 2025 Nov 3;12:1665813. doi: 10.3389/fnut.2025.1665813 (PMC12620502; doi:10.3389/fnut.2025.1665813)

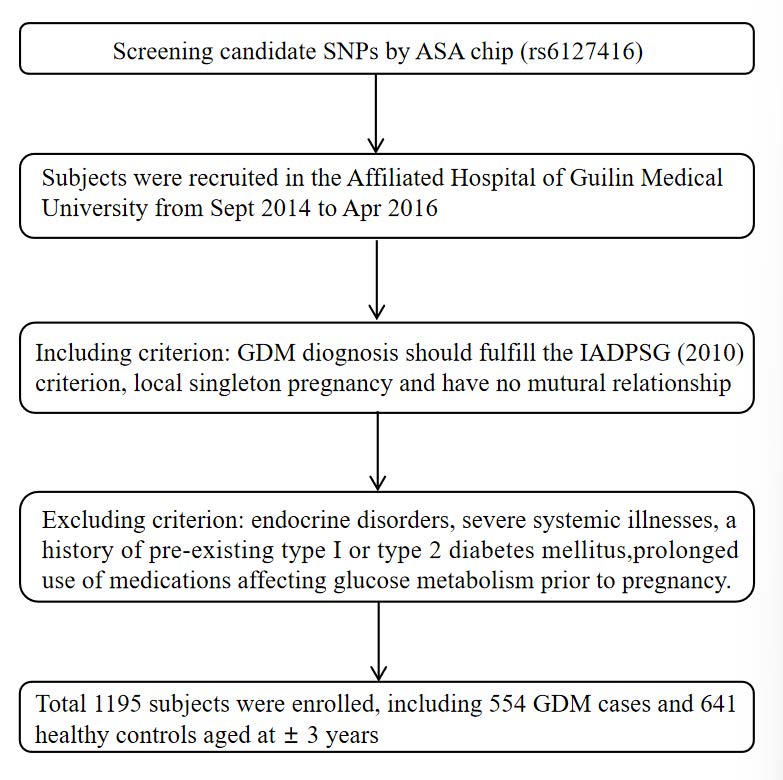

Supplement: Supplementary file 2 [file Image_1.jpg]

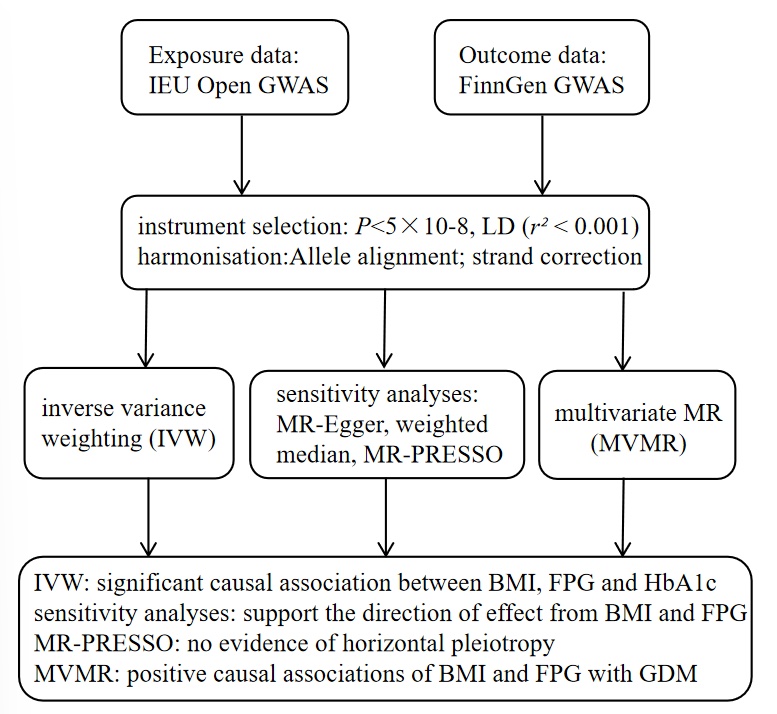

Supplement: Supplementary file 3 [file Image_2.jpg]
